# Identifying the knowledge structure of electromagnetic fields and health research: Text network analysis and topic modeling

**DOI:** 10.1371/journal.pone.0273005

**Published:** 2022-08-17

**Authors:** GyeongAe Seomun, Suyeon Ban, Jinkyung Park

**Affiliations:** 1 College of Nursing, Korea University, BK21FOUR R&E Center for Learning Health, Systems, Korea University, Seoul, Republic of Korea; 2 College of Nursing, Chonnam National University, Gwangju, Republic of Korea; Children’s Hospital of Eastern Ontario (CHEO), University of Ontario, CANADA

## Abstract

**Background:**

With technological and scientific advancement, people are being increasingly exposed to electromagnetic fields, particularly from portable devices such as mobile phones. However, there is currently no consensus regarding the health effects of electromagnetic field exposure, despite the large amount of research conducted on this topic. This study aimed to understand the knowledge structure and trend of electromagnetic field and health research through text network analysis and topic modeling.

**Methods:**

PubMed, Embase, and Cochrane were searched, and 3,880 articles published before June 2021 were identified. We explored the main keywords and research topics regarding electromagnetic fields and human health by constructing a network of keywords. A social network analysis program was used to analyze the data, visualize the network, and perform topic modeling.

**Results:**

Four keywords, “exposure,” “effect,” “cell,” and “cancer,” were highly correlated to other keywords and formed each colony in the knowledge structure of research on electromagnetic fields and health. Five topics were derived from topic modeling: cell research, research on the adaption of MRI, health effects of mobile phones, pain therapy, and exposure measurement. Cell research has been continuously performed, and many studies have been conducted on the health effects of mobile phones since 2000.

**Conclusions:**

These findings will assist in gaining insights into and understanding changes in research on the health effects of electromagnetic fields, and suggest important areas and directions for future research.

## Introduction

Electromagnetic radiations are produced by natural and anthropogenic sources [1], and owing to scientific and technological advancements, people are constantly exposed to electromagnetic fields (EMFs). Anthropogenic non-ionizing EMFs can be classified as extremely low-frequency (ELF) or radiofrequency (RF) range [2,3]. The electricity emitted from power sockets is associated with ELF-EMFs, whereas RF-EMFs (which have higher frequencies) are used for information transmission via TV antennas, radio stations, mobile phones, Wi-Fi, and fifth-generation (5G) technology [2].

The increasing use of wireless devices has increased public exposure to EMFs [4]. Therefore, international scientific organizations and regulatory agencies have investigated the potential health risks associated with EMF exposure and published guidelines for limiting public exposure to EMFs [4,5]. The International Commission on Non-Ionizing Radiation Protection (ICNIRP) and the International Committee for Electromagnetic Safety (ICES) have published guidelines for limiting public exposure to EMFs [5]. In 2002, the International Agency for Research on Cancer (IARC) classified ELF-EMFs as possibly carcinogenic to humans (Group 2B) due to limited clinical evidence, inadequate experimental support, and lack of plausible mechanisms at the exposure levels observed in epidemiological studies [1]. In 2011, the IARC also classified RF-EMFs into Group 2B for similar reasons as well as the need for additional long-term studies with adequate levels of RF-EMFs exposure [6].

Studies have been conducted on the potential health effects of EMFs, including epidemiologic studies, animal and cell research, and EMF exposure assessments. However, the effects of EMFs on the human body remain unclear, and the World Health Organization (WHO) has continuously investigated this by promoting the International EMF Project since 1996 [7]. To assess and manage possible EMF risks, the WHO has identified and recommended exploring relevant research topics. Furthermore, in 2019, the WHO prioritized research on possible health implications of increased mobile phone usage [8,9], and commissioned systematic reviews to examine and synthesize the available data. As a consensus on this subject has yet to be reached, previous studies should be analyzed to identify evidence [2,10].

Although systematic reviews and meta-analyses are commonly used for this, they are unsuitable for macro-analyses as they aim to identify answers to specific research questions [11]. Social network analysis (SNA) is an analytical method that can be applied to a large amount of data and is commonly used to identify the contextual meaning of words and their relationships. Texts can be analyzed by coding them into conceptual or semantic networks [12]. Text network analysis (TNA) can be used to analyze extensive text materials in big data using SNA [13]. Therefore, TNA can be used to identify knowledge structures and research topic trends based on the frequency, centrality, and co-occurrence of keywords [14]. Knowledge structure analysis using text networks quantitatively derives the key concepts of a particular field and helps visualize relationships between them [15]. By identifying the knowledge structure of EMFs and health research, we can identify research trends and suggest future research directions. Therefore, in this study, we conducted TNA to identify research themes and trends over time and investigate the properties of the resulting knowledge structure.

## Methods

We used TNA to explore the main keywords and research topics regarding EMFs and human health by constructing a network based on the co-occurrence of keywords in the associated literature. TNA encodes the relationships between words in a text and constructs a network of the linked words [14]. The relationships between keywords can be used to identify actual and potential knowledge structures and trends of a research topic [12]. Furthermore, based on the quantification value, network analysis can provide a more novel perspective on research topics than conventional methods [12,14].

### Data search and collection

**Fig 1:**
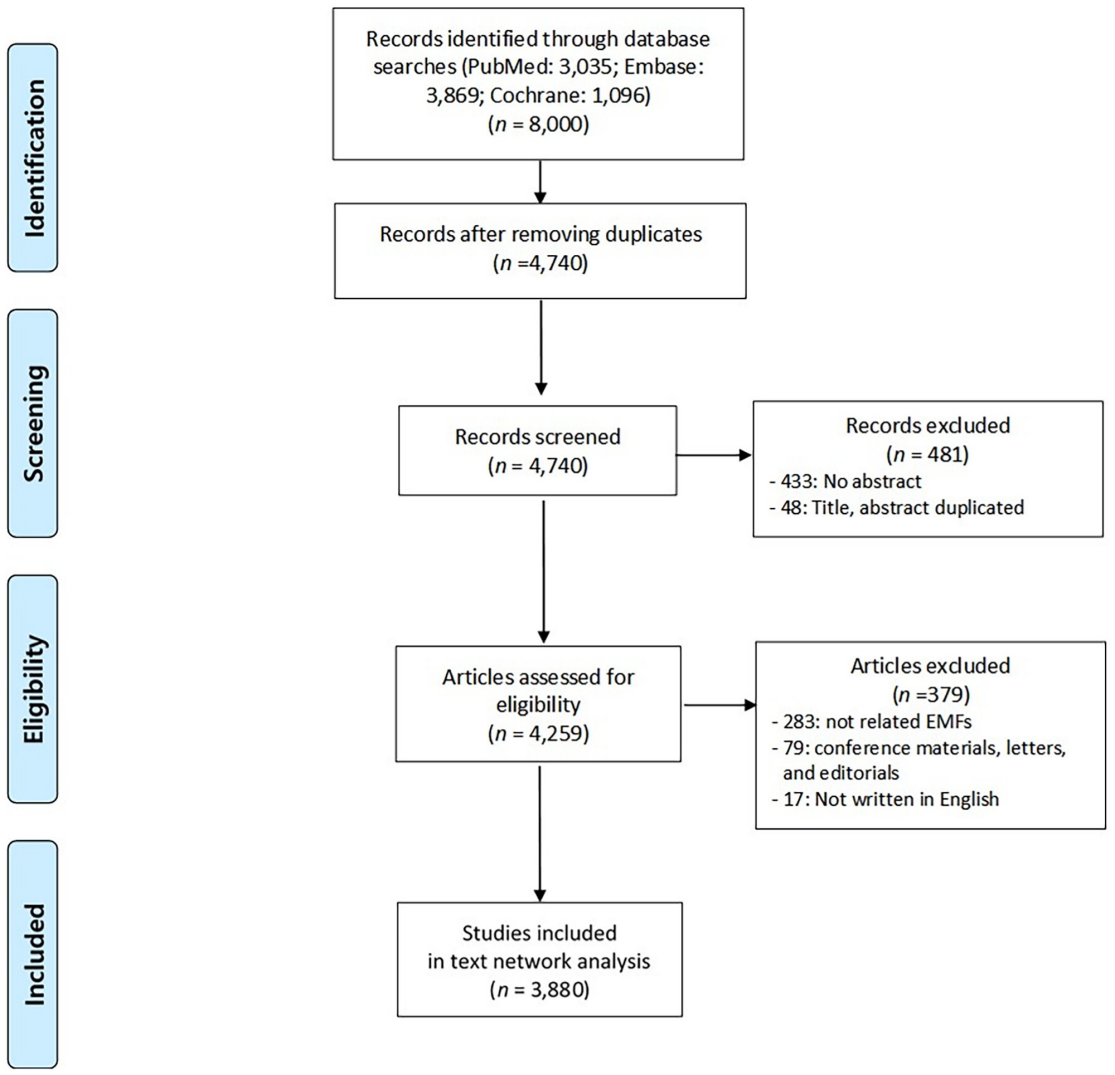
The flowchart of the article inclusion process in health effects of the electromagnetic fields. This shows the process from the search results in 3 databases to the selection of the final included articles.

PubMed, Embase, and the Cochrane Library databases were used to search for EMF-related literature in June 2021 using the related keyword of “electromagnetic field” in the title or abstract. (S1 Table). We limited the search to human research and manuscripts in English, excluding duplicates and articles without abstracts, to identify 3,880 studies (Fig 1). We identified vital information from these included studies using citation information from the databases and organized the information using a predefined Excel form (S2 Table).

To account for words expressed in different forms or repetitions, word refinement was performed before generating the keyword matrix. A dictionary including a thesaurus, defined words, and exception words was created. First, words and abbreviations with identical or similar meaning were grouped together and designated as one representative word. To prevent any meaning overlaps in the analysis, all words were changed to lowercase letters in singular form. Second, two or more morphemes were grouped and extracted as a single word. Finally, the exceptions list was created by determining morphemes to be excluded from the analysis (such as analysis terms and abstract types). The word “Electromagnetic field,” for which the literature search was conducted, was included in the “exceptions dictionary” as it was mentioned in all studies. We repeated the analysis of words in the abstract while creating the dictionary and decided on the words to be registered in the dictionary based on the advice of a text network analysis expert and a librarian.

### Generation of the keyword matrix and network

By applying the thesaurus, defined words, and exception-words dictionary, we identified 22,797 keywords and their frequency of appearance. In TNA, the main phenomena can be clearly identified by focusing on repetitive subject words and generally, only keywords appearing at a certain minimum frequency were included in the analysis [16]. In this study, words with a frequency of occurrence of 10 or more were identified as keywords, so that the top 10% of words were included in the analysis to represent the main content of the text.

In TNA, a node represents a keyword of a paper, and co-occurrence refers to the repeated occurrence of a keyword. The same keywords are present in different papers for facilitating the formation of links and networks. Therefore, we generated a matrix to evaluate the frequency of co-occurrence between previously selected keywords and constructed a network of keywords representing co-occurrence relationships with connecting links. Frequent occurrence of two words indicated that they presented similar associations and significant contextual relationships [14]. We generated a total of 64,409 one-mode matrices from two-mode matrices and analyzed studies at ten-year intervals to identify changes in EMF-research subjects over time.

### Keyword analysis and visualization

We analyzed the frequency and degree of closeness and betweenness centralities. These centrality indicators are commonly used in TNA [16] and are keyword quantification values. Centrality indicates the number of nodes centered in a network based on their relative ranking. Keywords with high centrality are considered main keywords. Degree centrality indicates a high incidence of connection with other research keywords, i.e., the level of influence between keywords. Closeness centrality measures the connection distance between nodes to indicate their proximity. Betweenness centrality measures the mediation level between keyword groups [16,17].

The knowledge structure was used to visualize the network structure, node, and connection strength to be included in the sociogram using keywords with high frequency and degree centrality [12]. The network data and analysis results were graphically visualized using NetMiner 4.0 (Cyram Inc., Seongnam, Korea).

### Topic modeling

Latent Dirichlet allocation (LDA) is the most frequently used algorithm in topic modeling that learns a set of topics from words that tend to occur together in documents [15]. It identifies hidden topics within documents and document sets and uncovers the ratio of topics for each document and the probability of each word being included in each topic [17].

We performed topic analysis using LDA and selected small Dirichlet hyper parameters, i.e., α = 0.1 (prior to per-document topic distribution) and β = 0.01 (prior to per-topic word distribution) to obtain a sparse topic and word distribution and, consequently, more interpretable topics. As it is difficult to select an optimal number of topics [18], we analyzed various topics and compared the similarity and difference of their contents using different models. We also performed this analysis based on different time periods to identify temporal changes.

## Results

### Keywords and knowledge structure of the EMFs and health

**Fig 2:**
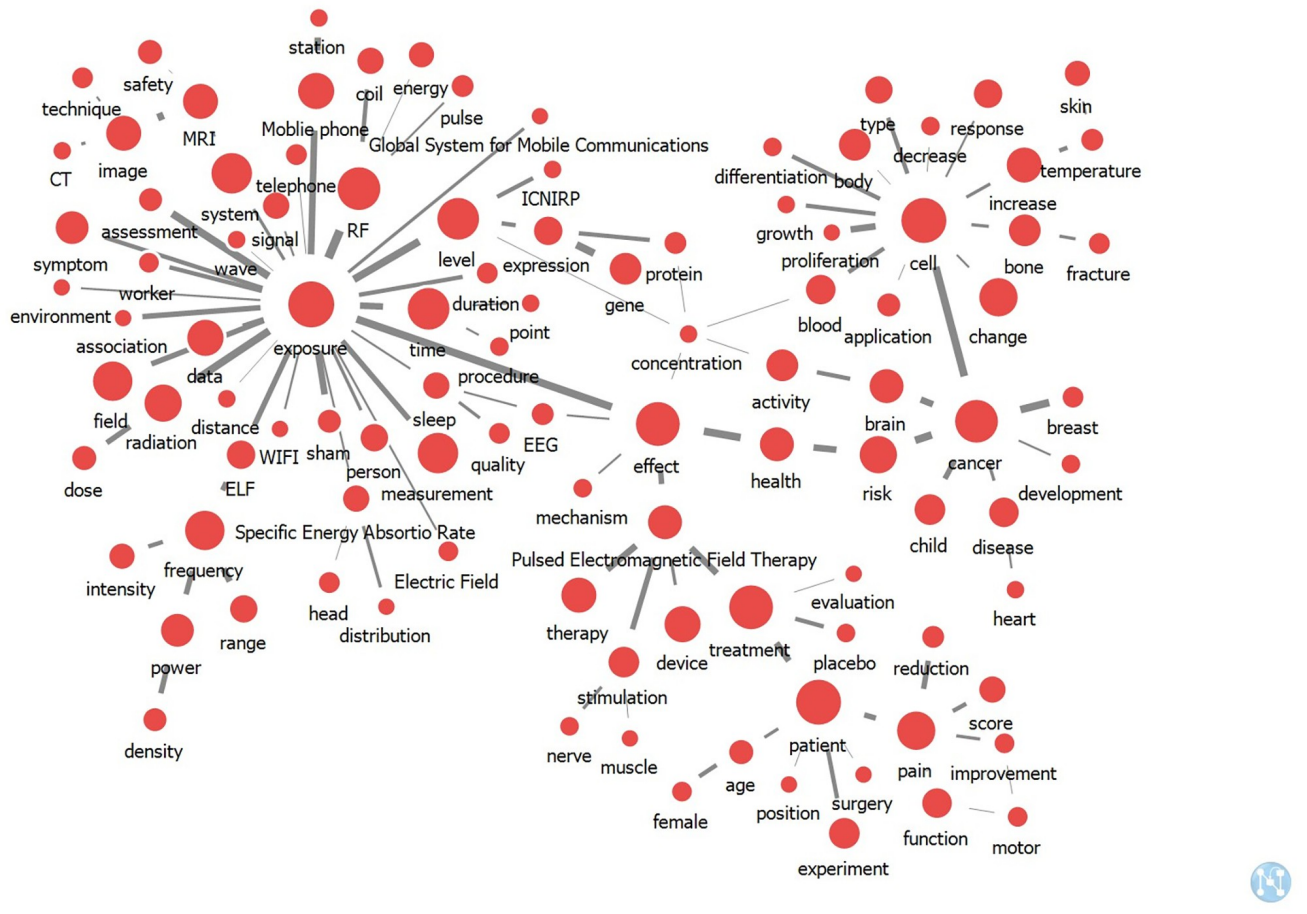
Overall keyword network structure for electromagnetic field and health research. The size of the node indicates the degree centrality of the keyword, and the width of the line indicates the strength of the link between keywords.

Table 1 shows the top 30 keywords by frequency and the three centrality indices. “Exposure,” “cell,” “patient,” “effect,” and “treatment” showed high frequency and centrality, suggesting that they appeared regularly (Table 1). The knowledge structure of EMFs and health was also identified. Four keywords (i.e., “exposure,” “effect,” “cell,” and “cancer”) were highly correlated to other keywords, and each colony was formed around these four keywords (Fig 2).

**Table 1:** Top 30 keywords that emerged from electromagnetic field and health research.

Rank	Keyword	Frequency	Keyword	Degree Centrality	Keyword	Betweenness Centrality
1	exposure	6561	exposure	0.1367	exposure	0.0891
2	effect	4111	patient	0.1168	cell	0.0747
3	cell	4078	cell	0.1149	patient	0.0741
4	patient	3932	effect	0.0905	effect	0.0388
5	treatment	2698	treatment	0.0766	treatment	0.0317
6	RF	2244	RF	0.0575	level	0.0205
7	cancer	1942	cancer	0.0552	system	0.0189
8	level	1930	level	0.0533	time	0.0183
9	measurement	1606	time	0.0507	cancer	0.0178
10	risk	1568	system	0.0484	RF	0.0177
11	time	1531	measurement	0.0473	measurement	0.0167
12	system	1451	frequency	0.0413	pain	0.0122
13	frequency	1439	field	0.0409	field	0.0118
14	radiation	1287	pain	0.0394	frequency	0.0116
15	Pulsed Electromagnetic Field Therapy	1284	change	0.0387	device	0.0112
16	Mobile phone	1281	radiation	0.0361	image	0.0102
17	pain	1269	risk	0.0349	MRI	0.0100
18	data	1268	device	0.0334	risk	0.0097
19	field	1251	Mobile phone	0.0330	gene	0.0095
20	therapy	1247	data	0.0315	power	0.0094
21	device	1149	image	0.0312	activity	0.0093
22	experiment	1099	increase	0.0312	radiation	0.0091
23	change	1074	therapy	0.0312	Mobile phone	0.0088
24	health	1053	MRI	0.0308	data	0.0088
25	MRI	1022	brain	0.0304	experiment	0.0087
26	image	1012	Pulsed Electromagnetic Field Therapy	0.0293	change	0.0082
27	stimulation	926	health	0.0293	health	0.0080
28	brain	856	power	0.0289	function	0.0074
29	gene	854	symptom	0.0282	bone	0.0073
30	increase	851	activity	0.0278	blood	0.0073

ELF = Extreme Low Frequency; MRI = Magnetic Resonance Imaging; RF = Radio Frequency.

### Trends in EMF and health research over time

From 1964 to June 2021, 3,880 articles on EMFs and health were published (S1 Fig). Since 2010, the number of related studies has increased considerably. Research trends were analyzed at 10-y intervals, and research before 1990 was considered a single period due to the limited number of articles. The keywords “exposure,” “cell,” “effect,” “patient,” and “cancer” ranked high throughout all periods. Prior to 1990 (136 studies), “bone,” “fracture,” “radiation,” “energy,” and “diathermy” emerged with higher centrality than in the other periods. In 1991–2000 (426 studies), “child,” “melatonin,” “leukemia,” “association,” and “worker” were the main keywords. In 2001–2010 (998 studies), “pain,” “RF,” and “mobile phone” emerged, and “pulsed electromagnetic field therapy” and “specific energy absorption rate” became significant centrality keywords. In 2011–2021 (2,320 studies), “data” emerged and “pulsed electromagnetic field therapy” remained with high centrality (Table 2).

**Table 2:** Top 30 keywords by degree centrality over time.

Degree centrality	≤1990	1990s	2000s	2010s
1	cell	exposure	exposure	exposure
2	exposure	cell	patient	patient
3	cancer	patient	cell	cell
4	effect	cancer	effect	effect
5	patient	effect	level	treatment
6	body	risk	treatment	RF
7	frequency	level	time	cancer
8	treatment	time	cancer	level
9	fracture	measurement	measurement	time
10	bone	field	frequency	system
11	field	child	pain	measurement
12	radiation	treatment	RF	pain
13	coil	system	field	MRI
14	blood	frequency	system	frequency
15	probe	increase	mobile phone	change
16	RF	change	risk	device
17	energy	power	change	radiation
18	MRI	brain	increase	risk
19	system	type	data	image
20	diathermy	leukemia	stimulation	mobile phone
21	flow	radiation	experiment	therapy
22	gene	activity	power	Pulsed Electromagnetic Field Therapy
23	heat	data	response	field
24	level	stimulation	brain	data
25	measurement	worker	activity	health
26	microwave	blood	Pulsed Electromagnetic Field Therapy	symptom
27	pulse	breast	Specific Energy Absorption Rate	increase
28	stimulation	sleep	health	bone
29	Pulsed Electromagnetic Field Therapy	gene	therapy	brain
30	activity	protein	ELF	body

ELF = Extreme Low Frequency; MRI = Magnetic Resonance Imaging; RF = Radio Frequency.

### Topic modeling of EMFs and health research

**Fig 3:**
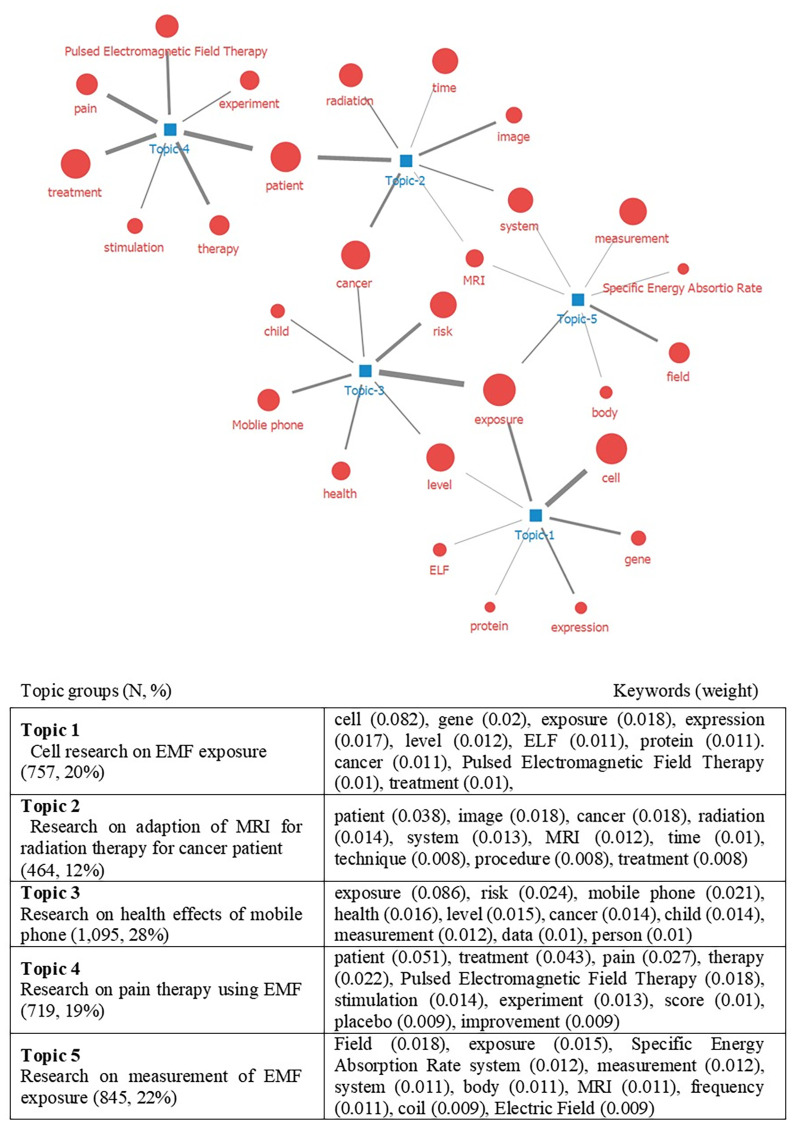
Topic modeling of electromagnetic field and health research. N indicates the number of main documents on each topic. The weight refers to the degree to which the keyword represents the topic.

LDA topic analysis identifies topics commonly included in the literature based on unsupervised learning. The topics are transformed into keyword combinations based on statistical results, and experts judge the meaning of the combinations and derive meaningful topics. Several rounds of LDA were performed on varying numbers of topics. After we grouped the subtopics through discussion, K = 5 topics with no overlapping meanings between groups were identified (Fig 2). Each topic was ranked with reference to word weight, and the top 10 collocates in the corresponding topic were extracted. We combined meaningful keywords to form topic groups and derived five groups (Fig 2). Topic 1 (cell research on EMF exposure) included “cell,” “gene,” “exposure,” “expression,” and “level.” Topic 2 (adaptation of magnetic resonance imaging (MRI) for radiation therapy (RT) applied to cancer patients) included “patient,” “image,” “cancer,” “radiation,” and “MRI.” Topic 3 (effects of EMFs from mobile phones) included “exposure,” “risk,” “mobile phone,” “health,” and “cancer.” Topic 4 (pain therapy using EMFs) included “patient,” “treatment,” “pain,” “therapy,” and “pulsed electromagnetic field therapy.” Topic 5 (measurement of EMF exposure) included “field,” “exposure,” “specific energy absorption rate,” “measurement,” “system,” and “body.” The network between keywords in each topic group is shown in Fig 3.

### Trends in the topics of EMFs and health research over time

To identify trends in the topics of EMFs and health research, we performed topic modeling based on time periods. Prior to 1990, topics largely included “cell,” “field,” “exposure,” “frequency,” and “body.” These cell research topics were reduced to 18% in the 1990s, when the most common topics included “exposure,” “cancer,” “risk,” “child,” and “leukemia.” In the 2000s, research topics included “exposure,” “specific energy absorption rate,” “mobile phone,” “system,” and “field.” From 2010 to June 2021, most EMF-related health research on mobile phones included “exposure,” “mobile phone,” “level,” “risk,” and “health” (S3 Table).

## Discussion

This study aimed to provide insights into EMF and health research by quantitative and qualitative analyses of the network of the main keywords in the published literatures. We present a scientific perspective on the subject obtained by observing the trends in research topics and identifying the knowledge structure of EMF and health research. Our results show the macro network centering on keywords of “exposure,” “effect,” “cell,” and “cancer” in the knowledge structure in EMF and health research. A keyword with high centrality can influence the research trend considerably and form a network upon connection with other keywords [13]. The study found that “exposure” was the center of a large-scale network analysis group, indicating that a lot of studies were performed around this network of keywords. The keywords of “exposure standards,” “mobile phone,” “Wi-Fi,” and “MRI” were also networked with “exposure.” The network group of the keyword “effect" is linked to “muscle,” “nerve,” “stimulation,” and “pulsed electromagnetic field therapy.” In addition, “cancer” was centered on studies related to both “child” and “brain,” and a strong network was formed with “cell.” Therefore, we confirmed that cancer research was widely performed using cell research.

Considering research trends over time, “bone,” “fracture,” “radiation,” “energy,” and “diathermy” were commonly observed before 1990. Several studies investigated the effects of pulsed EMFs on bone healing after the Food and Drug Administration approval in 1979 [19–21]. Research before 1990 also focused on local RF hyperthermia for tumor treatment, treatment modality design, and diathermy technique [22,23]. With the invention of MRI in the late 1970s, several studies addressed its safety, accuracy, and diagnostic capability [24].

“Child,” “melatonin,” “leukemia,” “association,” and “worker” were the main keywords in 1991–2000. Most studies in the 1990s, led by the WHO and ICNIRP, explored the health effects of ELF from residential power stations and home electronic appliances, and indicated a weak relation with childhood leukemia. Therefore, ELF was classified as a possible human carcinogen (Group 2B) by the IARC in 2002 [1,25]. Studies investigating adults in this period produced unclear results, particularly for the carcinogenic potential of occupational ELF exposure and nighttime exposure [26].

In 2001–2010, “pain,” “RF,” and “mobile phone” emerged, and “pulsed electromagnetic field therapy” and “specific energy absorption rate” became significant centrality keywords. In the 2000s, EMF studies started to focus on the relationship between RF exposure (mobile phones and wireless communication) and adverse health effects, particularly brain cancer. IARC classified RF as a possible human carcinogen (Group 2B) in 2011 based on a long-term epidemiological study [6,27].

In 2011–2021 (2,320 studies), “data” emerged as a keyword and “pulsed electromagnetic field therapy” continued to rank highly as a centrality keyword. After the 2000s, few studies investigated the therapeutic effects of electric current therapies. In the 2010s, several studies addressed the efficacy of pulsed EMFs for chronic pain from musculoskeletal disorders [28].

LDA was used to identify focus topics based on keywords. By categorizing the keywords, we derived the following five meaningful topic groups. Topic 1 consisted of keywords related to “cell research on EMF exposure”. “cell,” and “gene,” and “exposure” exhibited the highest frequency and centrality. As expected for epidemiologic studies, randomized controlled trials are limited to human health effects, particularly for cancer, because of ethical issues and long-exposure requirements. To overcome these issues, *in vitro* experiments are performed for examining the response of human cells and genes to ELF-EMFs [29].

Topic 2 was related to “adaption of MRI for RT of cancer patients.” Introducing MRI prior to RT as a treatment pathway has attracted interest as it provides improved soft-tissue image contrast, thereby enabling RT to be tailored and adapted to patients [30].

Topic 3 was associated with “health effects of EMFs from mobile phones.” In 2000, IARC coordinated a study in 13 countries for investigating whether RF-EMFs from mobile phones increase the risk of cancer [27,31]. Since 2011, large-scale epidemiological studies have been conducted on this subject. The MOBI-KIDS study, a multi-national epidemiological study, evaluated the potential carcinogenic effects of exposure to RF and ELF-EMFs from mobile phones on tumors on the central nervous system of children and adolescents [32]. Although a carcinogenic risk was not clearly identified, this study highlighted the need for further investigation on adverse effects to the central nervous system [33]. Other large-scale epidemiology studies include the Cohort Study of Mobile Phone Use and Health and the Advanced Research on Interaction Mechanisms of Electromagnetic Exposures with Organisms for Risk Assessment [34,35].

Epidemiological studies have primarily been performed for identifying long-term health impacts, including the causes of pediatric cancer, which have attracted public attention because of the vulnerability of children during their growth period [36].

Topic 4 comprised keywords associated with “pain therapy using EMF.” EMFs have been used for pain relief for decades, including pulsed EMFs for musculoskeletal pain or bone healing. Several studies have been conducted to identify the efficacy or effect of such usage [28,37].

Topic 5 was associated with “measurement of EMF exposure.” The measurement of EMF exposure using human modeling or field calculations is a major research topic, particularly for RF. Specific absorption rate (SAR) in the human head is the most common evaluation measure. For instance, the SAR of patients during MRI utilization, which generates RF, has been analyzed to investigate patient safety [38,39].

From analyzing the topic model over time, the EMF and health research topic trends could be confirmed. EMF-related cell research has been continuously conducted, but at a decreasing rate, and research on “pulsed electromagnetic field therapy” has been conducted as well. Since 2000, an increasingly large number of studies have investigated the health effects of mobile phones, in accordance with the WHO’s recommendations [8,9].

### Limitations

Our investigation was limited to human studies as the purpose was to establish the research trends of EMF effects on human health. As animal research was excluded, relevant studies regarding EMF effects on animals might have been excluded among them. Furthermore, the literature collection was limited to three databases. Nevertheless, they are representative international databases in the health field, so it can be assumed that most papers on EMFs and health were included. Methodological limitations included the use of only titles and abstracts to extract texts in TNA, so keywords with low frequency or centrality were excluded. Therefore, the generalization of these results requires reasoning and evidence.

## Conclusion

Using TNA, we investigated the research trends on EMFs and human health through several approaches. To the best of our knowledge, this is the first study to identify this knowledge structure. The relationship between research keywords, the structure of the central keyword for each topic, and changes in the main research topics over time were identified, and the trends and semantic networks of published studies were detailed. Future research directions can be inferred based on these findings.

Since 1996, WHO has organized the International EMF Project to promote intensive and high-quality research programs on EMF exposure and health risks. Several laboratory studies have been conducted on short-term effects, and epidemiologic studies have been conducted on long-term effects. In addition, large-scale multinational studies such as MOBI-Kids Study, INTEROCC study, and COSMOS have been conducted [32–35]. However, the IARC classified ELF and RF-EMFs as being possibly carcinogenic to humans (Group 2B) due to limited evidence of plausible mechanisms to explain the exposure levels observed in epidemiological studies. They suggested the need for additional long-term studies for identifying evidence of EMF exposure and health risks [1,6]. In this study, research on the health effects of mobile phones related to exposure to RF-EMFs and the measurement of EMF exposure were found to account for more than 50%. Additionally, the WHO EMF project has commissioned a systematic review study that synthesizes the results of available EMF studies on human effects in 2019 [8,9]. Therefore, both an integrated study and a large-scale epidemiologic study that can confirm high-level evidence on EMF exposure and health effects are needed.

## Supporting information

S1 Fig The number of the electromagnetic field articles from 1964—June 2021. (DOCX)

S1 Table Search strategy in the databases. (DOCX)

S2 Table The excel form of extraction from included studies. (DOCX)

S3 Table Trends in topics of the electromagnetic field and health research over time. (DOCX)

## References

[1] IARC Working Group on the evaluation of carcinogenic risks to humans. Non-ionizing radiation, part 1: static and extremely low-frequency (ELF) electric and magnetic fields. IARC Monographs on the Evaluation of Carcinogenic Risks to Humans 2002; 80:1–390. . 12071196 PMC5098132

[2] IARC Working Group on the evaluation of carcinogenic risks to humans. Non-ionizing radiation, Part 2: Radiofrequency electromagnetic fields. IARC monographs on the evaluation of carcinogenic risks to humans. World Health Organization, International Agency for Research on Cancer 2013; 102(Pt 2):1–460. 24772662. PMC4780878 24772662

[3] Kheifets LRM, Saunders R, van Deventer E. The sensitivity of children to electromagnetic fields. Pediatrics 2005;116(2):e303–13. doi: 10.1542/peds.2004-2541 . 16061584

[4] Kromhout HSP, Huss A, van Nierop LE, Bongers S, Schaap K, de Vocht F. ICNIRP statement on diagnostic devices using non-ionizing radiation: Existing regulations and potential health risks. Health Phys. 2017;113(2):305–321. doi: 10.1097/HP.0000000000000686 . 28658061

[5] ICNIRP statement on diagnostic devices using non-ionizing radiation: Existing regulations and potential health risks. Health Phys. 2017;112(3):305–21. doi: 10.1097/HP.0000000000000654 28121732 PMC5515634

[6] World Health Organization. IARC Classifies Radiofrequency Electromagnetic Fields as Possibly Carcinogenic to Humans. Lyon: IARC; 2011. https://www.iarc.who.int/wp-content/uploads/2018/07/pr208_E.pdf.

[7] World Health Organization. The International EMF Project: Health and environmental effects of exposure to static and time varying electric and magnetic fields: minutes of the Second International Advisory Committee Meeting, 2–3 June 1997, Geneva: World Health Organization; 1997. https://apps.who.int/iris/handle/10665/63860?locale-attribute=de&.

[8] World Health Organization. WHO Research Agenda for Radiofrequency Fields. 2010. ISBN: 9789241599948. https://apps.who.int/iris/handle/10665/44396.

[9] World Health Organization. Call for Expressions of Interest for Systematic Reviews; 2019. https://www.who.int/news-room/articles-detail/call-for-expressions-of-interest-for-systematic-reviews-(2019).

[10] Hardell L. World Health Organization, radiofrequency radiation and health—a hard nut to crack (Review). Int J Oncol. 2017;51(2):405–413. doi: 10.3892/ijo.2017.4046 . 28656257 PMC5504984

[11] Bartolucci AA, Hillegass WB. Overview, strengths, and limitations of systematic reviews and meta-analyses. In: Chiapelli F, editor. Evidence-based practice: Toward optimizing clinical outcomes. Berlin: Springer; 2010. p. 17–33.

[12] Drieger P. Semantic network analysis as a method for visual text analytics. Procedia-Social Behav Sci. 2013;79:4–17. doi: 10.1016/j.sbspro.2013.05.053

[13] Ding Y, Chowdhury GG, Foo S. Bibliometric cartography of information retrieval research by using co-word analysis. Info Proc Manage. 2001;37(6):817–42. doi: 10.1016/S0306-4573(00)00051-0

[14] He Q. Knowledge discovery through co-word analysis. Libra Trends 1999;48:133–159.

[15] Valdez D, Picket AC, Young B-R, Golden S. On mining words: The utility of topic models in health education research and practice. Health Promo Prac. 2021;22(3):309–12. doi: 10.1177/1524839921999050 . 33759597

[16] Lee S-S. A content analysis of journal articles using the language network analysis methods. J Korean Soc Info Manage. 2014;31(4):49–68.

[17] Zhang J, Luo Y, editors. Degree centrality, betweenness centrality, and closeness centrality in social network. Proceedings of the 2017 2nd International Conference on Modelling, Simulation and Applied Mathematics (MSAM2017); 2017 Mar 26–27; Bangkok, Thailand. Dordrecht: Atlantic Press; 2017.

[18] Park J-H, Song M. A study on the research trends in library & information science in Korea using topic modeling. J Korean Soc Info Manage. 2013;30(1):7–32.

[19] Wang B, Liu Y, Liu Z, Li M, Qi M, editors. Topic selection in latent dirichlet allocation. 11th International Conference on Fuzzy Systems and Knowledge Discovery (FSKD); 2014 Aug 19–21; Xiamen, China. IEEE; 2014.

[20] Bassett CAL. The development and application of pulsed electromagnetic fields (PEMFs) for ununited fractures and arthrodeses. Ortho Clinics North Am. 1984;15(1):61–87. . 6607442

[21] Sneed NV, VanBree KM. Treating ununited fractures with electricity: nursing implications. J Gerontol Nurs. 1990;16(8):26–31. doi: 10.3928/0098-9134-19900801-08 2387968

[22] Chou C, Ren R. Radio frequency hyperthermia in cancer therapy. In: Bronzino J, editor. The Biomedical Engineering Handbook, 2nd ed. CRC Press; 1995. p. 93-1–93-5.

[23] Lerch IA, Kohn S. Radiofrequency hyperthermia: the design of coil transducers for local heating. Int J Radiat Oncol Biol Phys. 1983;9(6):939–48. doi: 10.1016/0360-3016(83)90022-6 . 6863066

[24] Mathur-De Vre R. Safety aspects of magnetic resonance imaging and magnetic resonance spectroscopy applications in medicine and biology: I. Biomagnetic effects. Arch Belg. 1987;45(9–10):394–424. . 3331256

[25] Angelillo I, Villari P. Residential exposure to electromagnetic fields and childhood leukaemia: A meta-analysis. Bull World Health Organ. 1999;77(11):906. . 10612886 PMC2557764

[26] Brainard GC, Kavet R, Kheifets LI. The relationship between electromagnetic field and light exposures to melatonin and breast cancer risk: a review of the relevant literature. J Pineal Res. 1999;26(2):65–100. doi: 10.1111/j.1600-079x.1999.tb00568.x . 10100735

[27] Khurana VG, Teo C, Kundi M, Hardell L, Carlberg M. Cell phones and brain tumors: A review including the long-term epidemiologic data. Surg Neurol. 2009;72(3):205–14. doi: 10.1016/j.surneu.2009.01.019 . 19328536

[28] Yang XH, Ye W, Perry TA, He C. Effects of pulsed electromagnetic field therapy on pain, stiffness, physical function, and quality of life in patients with osteoarthritis: A systematic review and meta-analysis of randomized placebo-controlled trials. Phys Ther. 2020;100(7):1118–31. doi: 10.1093/ptj/pzaa054 . 32251502

[29] Vijayalaxmi Prihoda TJ. Genetic damage in mammalian somatic cells exposed to extremely low frequency electro-magnetic fields: a meta-analysis of data from 87 publications (1990–2007). Int J Radiat Biol. 2009;85(3):196–213. doi: 10.1080/09553000902748575 . 19296340

[30] Woo S, Han S, Kim T-H, Suh CH, Westphalen AC, Hricak H, et al. Prognostic value of pretreatment MRI in patients with prostate cancer treated with radiation therapy: A systematic review and meta-analysis. Am J Roentgenol. 2020;214(3):597–604. doi: 10.2214/AJR.19.21836 . 31799874 PMC7499902

[31] Vila J, Bowman JD, Kincl L, Conover DL. Development of a source-based approach to assessing occupational exposure to electromagnetic fields in the INTEROCC study. Occup Environ Med. 2014;71:A35.3–A3A36. doi: 10.1136/oemed-2014-102362.110

[32] Turner MCG-LE, Momoli F, Langer CE, Castaño-Vinyals G, Kundi M, Maule M, et al. Nonparticipation selection bias in the MOBI-Kids study. Epidemiol. 2019;30(1):145–53. doi: 10.1097/EDE.0000000000000932 30299406 PMC6276861

[33] Calderón CIH, Taki M, Wake K, Addison D, Mee T, Maslanyj M, et al. ELF exposure from mobile and cordless phones for the epidemiological MOBI-Kids study. Environ Int. 2017;101:59–69. doi: 10.1016/j.envint.2017.01.005 . 28126406

[34] Schüz J, Dasenbrock C, Ravazzani P, Röösli M, Schär P, Bounds PL, et al. Extremely low‐frequency magnetic fields and risk of childhood leukemia: A risk assessment by the ARIMMORA consortium. Bioelectromagnetics 2016;37(3):183–9. doi: 10.1002/bem.21963 . 26991812

[35] Tettamanti GA, Åkerstedt A., Kojo T., Ahlbom K., Heinävaara A., Elliott S., et al. Long-term effect of mobile phone use on sleep quality: Results from the cohort study of mobile phone use and health (COSMOS). Environ Int. 2020;140:105687. doi: 10.1016/j.envint.2020.105687 . 32276731 PMC7272128

[36] Seomun G, Lee J, Park J. Exposure to extremely low-frequency magnetic fields and childhood cancer: A systematic review and meta-analysis. PloS One 2021;16(5):e0251628. doi: 10.1371/journal.pone.0251628 . 33989337 PMC8121331

[37] Peng L, Fu C, Xiong F, Zhang Q, Liang Z, Chen L, et al. Effectiveness of pulsed electromagnetic fields on bone healing: A systematic review and meta-analysis of randomized controlled trials. Bioelectromagnetics 2020;41(5):323–37. doi: 10.1002/bem.22271 . 32495506

[38] Golestanirad L, Iacono MI, Keil B, Angelone LM, Bonmassar G, Fox MD, et al. Construction and modeling of a reconfigurable MRI coil for lowering SAR in patients with deep brain stimulation implants. Neuroimage 2017;147:577–88. doi: 10.1016/j.neuroimage.2016.12.056 . 28011252 PMC5303648

[39] Martens AL, Slottje P, Meima MY, Beekhuizen J, Timmermans D, Kromhout H, et al. Residential exposure to RF-EMF from mobile phone base stations: Model predictions versus personal and home measurements. Sci Total Environ. 2016;550:987–93. doi: 10.1016/j.scitotenv.2016.01.194 . 26851884

