# Supplementary material for: Identifying the knowledge structure of electromagnetic fields and health research: Text network analysis and topic modeling
Source: PLoS One. 2022 Aug 17;17(8):e0273005. doi: 10.1371/journal.pone.0273005 (PMC9384997; doi:10.1371/journal.pone.0273005)
Supplement: S1 Fig — (DOCX) [file pone.0273005.s001.docx]

**S3 Figure** The number of the electromagnetic field articles from 1964 – June 2021
