# Supplementary material for: Identifying the knowledge structure of electromagnetic fields and health research: Text network analysis and topic modeling
Source: PLoS One. 2022 Aug 17;17(8):e0273005. doi: 10.1371/journal.pone.0273005 (PMC9384997; doi:10.1371/journal.pone.0273005)
Supplement: S1 Table — (DOCX) [file pone.0273005.s002.docx]

|  |  | **Search query** | **Results** |
| --- | --- | --- | --- |
| PubMed | 7 | ("electromagnetic field"[Title/Abstract] OR "non ionizing radiation"[Title/Abstract] OR "electro magnetic field"[Title/Abstract] OR "emf"[Title/Abstract] OR "non ionizing radiation"[Title/Abstract]) AND ((humans[Filter]) AND (english[Filter])) | **3,035** |
|  | 6 | "electromagnetic field"[Title/Abstract] OR "non ionizing radiation"[Title/Abstract] OR "electro magnetic field"[Title/Abstract] OR "emf"[Title/Abstract] OR "non ionizing radiation"[Title/Abstract] | 8,882 |
|  | 5 | "non ionizing radiation"[Title/Abstract] | 685 |
|  | 4 | "emf"[Title/Abstract] | 3,350 |
|  | 3 | "electro magnetic field"[Title/Abstract] | 69 |
|  | 2 | "non ionizing radiation"[Title/Abstract] | 685 |
|  | 1 | "electromagnetic field"[Title/Abstract] | 5,964 |
| Embase | #7 | (#1 OR #2 OR #3 OR #4 OR #5) AND [english]/lim AND [humans]/lim | **3,859** |
|  | #6 | #1 OR #2 OR #3 OR #4 OR #5 | 8,914 |
|  | #5 | 'non ionizing radiation':ab,ti | 760 |
|  | #4 | 'emf':ab,ti | 3,855 |
|  | #3 | 'electro-magnetic field':ab,ti | 82 |
|  | #2 | 'non-ionizing radiation':ab,ti | 760 |
|  | #1 | 'electromagnetic field':ab,ti | 5,563 |
| Cochrane | #1 | (electromagnetic field):ti,ab,kw in Cochrane Reviews, Trials (Word variations have been searched) | 965 |
|  | #2 | (non-ionizing radiation):ti,ab,kw | 14 |
|  | #3 | (EMF):ti,ab,kw | 138 |
|  | #4 | (electro-magnetic field):ti,ab,kw | 18 |
|  | #5 | (non ionizing radiation):ti,ab,kw | 104 |
|  | #6 | #1 OR #2 OR #3 OR #4 OR #5 | **1,096**  (review 17, Trials 1079) |

**S1Table** Search strategy in the database
