# Supplementary material for: Identifying the knowledge structure of electromagnetic fields and health research: Text network analysis and topic modeling
Source: PLoS One. 2022 Aug 17;17(8):e0273005. doi: 10.1371/journal.pone.0273005 (PMC9384997; doi:10.1371/journal.pone.0273005)
Supplement: S2 Table — (DOCX) [file pone.0273005.s003.docx]

**S2 Table** The excel form of extraction from included studies

| Author | Year | Title | Journal | Volume | Issue | Date | Type of Article | ISSN | DOI | Keywords | Abstract | URL |
| --- | --- | --- | --- | --- | --- | --- | --- | --- | --- | --- | --- | --- |
|  |  |  |  |  |  |  |  |  |  |  |  |  |
|  |  |  |  |  |  |  |  |  |  |  |  |  |
|  |  |  |  |  |  |  |  |  |  |  |  |  |
|  |  |  |  |  |  |  |  |  |  |  |  |  |
