# Supplementary material for: Identifying the knowledge structure of electromagnetic fields and health research: Text network analysis and topic modeling
Source: PLoS One. 2022 Aug 17;17(8):e0273005. doi: 10.1371/journal.pone.0273005 (PMC9384997; doi:10.1371/journal.pone.0273005)
Supplement: S3 Table — (DOCX) [file pone.0273005.s004.docx]

**S4 Table** Trends in topics of the electromagnetic field and health research over time

| Period | **Topic 1** | | **Topic 2** | | **Topic 3** | | **Topic 4** | | **Topic 5** | |
| --- | --- | --- | --- | --- | --- | --- | --- | --- | --- | --- |
|  | keyword | weight | keyword | weight | keyword | weight | Keyword | weight | keyword | weight |
| **≤1990** | radiation | 0.036 | exposure | 0.061 | cell | 0.069 | Patient | 0.077 | cell | 0.028 |
|  | energy | 0.025 | cancer | 0.049 | field | 0.039 | Fracture | 0.039 | membrane | 0.02 |
|  | pacemaker | 0.019 | risk | 0.017 | exposure | 0.024 | Treatment | 0.036 | pigment | 0.013 |
|  | coil | 0.018 | MRI | 0.013 | frequency | 0.02 | Bone | 0.019 | Ca2 | 0.011 |
|  | beam | 0.016 | function | 0.012 | body | 0.019 | Pulsed Electromagnetic Field Therapy | 0.017 | assay | 0.01 |
|  | 16% (22) | | 17% (23) | | 32% (44) | | 24% (32) | | 11% (15) | |
| **1990s** | exposure | 0.086 | system | 0.014 | exposure | 0.049 | Patient | 0.077 | cell | 0.103 |
|  | cancer | 0.058 | image | 0.013 | field | 0.022 | Treatment | 0.044 | exposure | 0.035 |
|  | risk | 0.051 | body | 0.012 | time | 0.018 | Therapy | 0.019 | activity | 0.018 |
|  | child | 0.028 | measurement | 0.012 | frequency | 0.017 | Symptom | 0.016 | gene | 0.017 |
|  | leukemia | 0.023 | coil | 0.012 | power | 0.015 | stimulation | 0.016 | level | 0.017 |
|  | 24% (102) | | 19% (81) | | 19% (79) | | 21% (88) | | 18% (76) | |
| **2000s** | exposure | 0.054 | exposure | 0.021 | exposure | 0.084 | Patient | 0.059 | cell | 0.079 |
|  | cancer | 0.05 | Specific Energy Absorption Rate | 0.019 | health | 0.025 | Treatment | 0.041 | exposure | 0.022 |
|  | risk | 0.033 | mobile phone | 0.015 | mobile phone | 0.022 | Pain | 0.029 | gene | 0.022 |
|  | ELF | 0.019 | system | 0.015 | level | 0.02 | stimulation | 0.02 | expression | 0.017 |
|  | child | 0.016 | field | 0.014 | measurement | 0.019 | Therapy | 0.019 | level | 0.015 |
|  | 14% (140) | | 27% (267) | | 21% (208) | | 21% (211) | | 17% (172) | |
| **2010s** | MRI | 0.019 | cell | 0.08 | patient | 0.048 | exposure | 0.088 | treatment | 0.043 |
|  | system | 0.018 | gene | 0.019 | cancer | 0.021 | mobile phone | 0.021 | patient | 0.043 |
|  | image | 0.018 | expression | 0.017 | radiation | 0.017 | level | 0.02 | pain | 0.029 |
|  | field | 0.014 | cancer | 0.013 | time | 0.011 | risk | 0.019 | therapy | 0.024 |
|  | body | 0.01 | exposure | 0.013 | treatment | 0.01 | health | 0.016 | Pulsed Electromagnetic Field Therapy | 0.021 |
|  | 20% (462) | | 21% (483) | | 12% (287) | | 29% (681) | | 18% (407) | |
